# Is urine microscopy a useful early biomarker for cardiac surgeryrelated AKI?

**DOI:** 10.1590/2175-8239-JBN-2019-0208

**Published:** 2020-02-21

**Authors:** Mark A. Perazella

**Affiliations:** 1 Yale University School of Medicine, Section of Nephrology, New Haven, CT, United States.

Urine microscopy with examination of the spun urine sediment is an invaluable diagnostic test for patients suspected of having kidney disease^1^^,^ 2. It is logical that injury to various nephron segments could be detected on examination of the urine sediment for cells and casts indicative of the site of injury. Along with clinical assessment, directed serum tests, and kidney imaging, urine microscopy allows the clinician to construct a rational differential diagnosis of the underlying kidney disease. It is particularly helpful in patients with acute kidney injury (AKI), hematuria, and proteinuria^1^^,^ 2.

Provider-performed urine microscopy often provides information that cannot be otherwise obtained by central laboratory urinalysis. Expert differentiation of urinary cell morphology, accurate identification of cellular and non-cellular casts, and recognition of various urinary crystals is akin to a “liquid kidney biopsy” ^(^ 1 ^,^ 2. In recent times, however, automated urine technology has been replacing urine sediment examination at many centers^1^^,^ 2. Also, identifying novel urine biomarkers of kidney disease has become a research priority in nephrology^1^^,^ 2. While the search for new tests that more accurately diagnose kidney disease is admirable, in my opinion microscopic examination of the urine sediment remains a valuable tool that should be preserved. Urine microscopy is a time-tested biomarker of kidney disease that has an important role in clinical nephrology.

Urine microscopy not only identifies AKI occurrence, but also provides more granular information into the nephron site of injury (e.g., glomerular, tubular or interstitial)^1^^,^ 2. For example, renal tubular cells (RTECs), RTEC casts, and muddy brown casts point to ischemic and/or nephrotoxic tubular injury. Dysmorphic erythrocytes and erythrocyte casts along with dipstick albuminuria typically indicate of glomerular injury, while culture negative pyuria, along with RTECs, WBC casts, and granular casts suggest acute tubulointerstitial disease in the proper clinical setting.

Urine microscopy also provides diagnostically useful information that can differentiate AKI that is due to prerenal azotemia from true renal parenchymal injury. In the appropriate clinical context, bland urine sediment with little cellular activity and primarily hyaline or few finely granular casts suggests AKI is due to a functional decline in GFR from renal hypoperfusion^3^. In contrast, urine sediment containing RTECs, RTEC casts, and coarse granular/muddy brown casts bespeaks structural injury from acute tubular injury (ATI), the most common cause of hospital-acquired AKI. This information helps to inform the clinician about the diagnosis and pathway of treatment to follow.

The utility of a urine sediment score based on RTECs and granular casts was demonstrated in 231 patients with hospital-acquired AKI from either prerenal azotemia or ATI^4^. A dose-dependent increase in likelihood ratios (LRs) for ATI was seen as the number of RTECs or granular casts increased, while the LRs declined for prerenal azotemia. The odds ratios (ORs) for ATI in patients with urine microscopy scores of 2 or greater versus 1 (no casts or RTECs) were 9.7 and 74, respectively. A pre-microscopy diagnosis of ATI with granular casts or urine sediment score ³2 had a positive predictive value of 100% for ATI. A pre-microscopy diagnosis of prerenal azotemia without RTECs or granular casts had a negative predictive value of 91% for prerenal AKI. Thus, urine microscopy is useful to differentiate these common causes of hospital-acquired AKI.

Urine microscopy can also predict important clinical end points. In the studies described in Table 1, urine microscopy predicted various AKI endpoints, which included worsening of kidney function as defined by higher AKIN stage, requirement for dialysis, and death^5^^-^ 9. Urine microscopy also compared favorably to novel biomarkers tested in some of the studies^7^^-^ 9. Risk classification of AKI determined by net reclassification index and integrated discrimination improvement was significantly improved after adding either urine microscopy or novel biomarkers to standard clinical variables. Thus, urine microscopy appears to not only have utility in differentiating the causes of AKI, but also predicting severity of AKI and death and improving upon baseline clinical determination of prognosis in hospital-acquired AKI.

**Table 1:** Urine microscopy utility for prognosis in AKI patients

Study (year)	Population	Patients (n)	Urine Scoring System	Clinical Outcomes	Findings
Chawla 2008 5	AKI on Nephrology service	18	Grade 1-4*	Non-recovery of kidney function	AUC 0.79
Perazella 2010 6	AKI on Nephrology service	197	Score 0 to ≥ 3^†^	Worsened AKI (increase in AKIN stage, KRT, or death)	AUC 0.75
					Score 1: RR 3.4
					Score 2: RR 6.6
					Score ≥3: RR 7.3
Hall 2011^7^	Patients with ≥ Stage 1 AKI	249	Score 0 to ≥ 3^†^	Worsened AKI (increase in AKIN stage, KRT, or death)	AUC 0.66
					Score 1: RR 1.6
					Score 2: RR 2.3
					Score ≥3: RR 3.5
Bagshaw 2012 8	ICU Patients with AKI	83	Score 0 to ≥ 3^$^	A. Worsened AKI	AUC 0.85
				B. KRT/death	Score 1-2: OR 5.6
					Score ≥3: OR 8.0
Schinstock 2012 9	ED patients	363	Any RTECs or RTECs/granular casts	AKIN Stages	AUC 0.58; Specificity for AKI 91%; Sensitivity 22%

* Grade 1: no casts or RTECs; Grade 2: at least 1 cast or RTECs seen but <10% of LPFs; Grade 3: many casts and RTECs seen on >10% but <90% of LPFs; Grade 4: sheets of muddy brown casts, casts and RTECs seen on >90% of LPFs.

† 0 casts or 0 RTECs, 0 points; 1-5 casts/LPF or 1-5 RTECs/HPF, 1 point each; ≥6 casts/LPF or ≥6 RTECs/HPF, 2 points each

$ 0 casts or 0 RTECs, 0 points; 1 cast or 1 RTEC/HPF, 1 point each; 2-4 casts or RTECs/HPF, 2 points each; ≥5 casts or RTECs/HPF, 3 points each Abbreviations: AKI- acute kidney injury, AKIN- Acute Kidney Injury Network, KRT- kidney replacement therapy, SCr- serum creatinine, RTEC- renal tubular epithelial cell, LPF- low power field, AUC- area under the curve, RR- relative risk, OR- odds ratio, ICU- intensive care unit, ED- emergency department.

In this issue of the Brazilian Journal of Nephrology, Goldani and colleagues examine the utility of urine microscopy (urine sediment score based on RTE cells and granular casts) in identifying AKI in patients undergoing cardiac surgery^10^. One hundred fourteen patients who underwent cardiac surgery had urine microscopy performed within the next 24 hours. Using KDIGO AKI criteria, the authors identified 23 patients (~20%) with AKI using serum creatinine criteria and 76 patients (~67%) using urine output criteria. Urine microscopy was highly specific in predicting AKI (~87% and ~92%, respectively); however, the test was insensitive (~35% and ~24%, respectively). The authors concluded that urine microscopy is highly specific for an early diagnosis of AKI in patients undergoing cardiac surgery. This study confirms previous findings and extends the utility of urine microscopy for diagnosing AKI early following cardiac surgery.

One of the major negatives of this study is the low sensitivity of urine microscopy in identifying AKI, a finding noted in other studies. It is likely that a number of AKI patients in this study had prerenal azotemia as the majority of AKI was stage I, which recovered back to baseline in 24 hours. One would expect the urine sediment in these patients to be bland. Identifying these patients and separating those from patients with higher stage or persistent AKI (>48 hours) would have likely improved the sensitivity of the test, but may have reduced specificity.

In summary, the authors are to be applauded for performing this study and adding to the literature supporting the continued use of rigorous examination of the spun urine sediment in the evaluation of patient with or at risk for AKI.
